# A phase 1b study of andecaliximab in combination with S-1 plus platinum in Japanese patients with gastric adenocarcinoma

**DOI:** 10.1038/s41598-022-13801-1

**Published:** 2022-06-30

**Authors:** Akira Ooki, Taroh Satoh, Kei Muro, Atsuo Takashima, Shigenori Kadowaki, Daisuke Sakai, Takashi Ichimura, Seiichiro Mitani, Toshihiro Kudo, Keisho Chin, Shigehisa Kitano, Dung Thai, Marianna Zavodovskaya, JieJane Liu, Narikazu Boku, Kensei Yamaguchi

**Affiliations:** 1 Department of Gastroenterological Chemotherapy, Cancer Institute Hospital of Japanese Foundation for Cancer Research, 3-8-31 Ariake, Koto-ku, Tokyo, 135-8550 Japan; 2 Palliative and Supportive Care Center, Osaka University Hospital, Osaka, Japan; 3 Department of Clinical Oncology, Aichi Cancer Center Hospital, Nagoya, Japan; 4 Division of Gastrointestinal Medical Oncology, National Cancer Center Hospital, Tokyo, Japan; 5 Department of Medical Oncology, Osaka International Cancer Institute, Osaka, Japan; 6 Department of Experimental Therapeutics, National Cancer Center Hospital, Tokyo, Japan; 7 Gilead Sciences, Inc., Foster City, CA USA

**Keywords:** Cancer, Drug discovery, Gastroenterology, Oncology

## Abstract

Andecaliximab (ADX) is a monoclonal antibody that inhibits matrix metalloproteinase 9 (MMP9), an extracellular enzyme involved in matrix remodeling, tumor growth, and metastasis. In preclinical models, MMP9 inhibitors have been shown to enhance the cytotoxic effects of chemotherapeutic agents and to suppress distant metastasis. In this phase Ib, multicenter study, the safety and efficacy of ADX combined with S-1 plus cisplatin (SP) or S-1 plus oxaliplatin (SOX) as a first-line treatment were evaluated in Japanese patients with advanced gastric or gastroesophageal junction (GEJ) adenocarcinoma. ADX was administrated at a dose of 800 mg every 2 weeks for the SP cohort and 1200 mg every three weeks for the SOX cohort. As of December 2019, 16 patients were enrolled (six patients in the SP cohort and 10 patients in the SOX cohort). Peripheral sensory neuropathy (69%), anorexia (63%), nausea (56%), and decreased neutrophil counts (44%) were the most common adverse events (AEs). The grade 3 or higher AEs attributed to ADX were stomatitis and abnormal hepatic function (each one patient) in the SP cohort and decreased neutrophil counts (two patients) in the SOX cohort. The objective response rate in 11 patients with measurable target lesions was 73% (8/11), based on the investigator’s evaluation. Median progression-free survival was11.9 months (90% confidence interval, 5.6–16.6), and median overall survival was not reached. In conclusion, ADX combined with S-1 plus platinum demonstrated a manageable safety profile and promising clinical activity in the first-line treatment of patients with advanced gastric or GEJ adenocarcinoma.

**Clinical Trial Registration information:** ClinicalTrials.gov Identifier: NCT02862535 (11/08/2016) and protocol ID: GS-US-296-1884.

## Introduction

Gastric or gastroesophageal junction (GEJ) adenocarcinoma remains a major clinical challenge, as it is the fifth most common cancer and the third leading cause of cancer-related deaths worldwide^1^. It is still frequently diagnosed in unresectable, advanced disease stages^2^, and systemic chemotherapy is the main therapeutic option for such patients^3,4^. However, the clinical benefits are limited, considering the five-year overall survival (OS) rate of 5–20%^5^. Therefore, further development of novel agents is required.

The extracellular matrix (ECM) regulates tissue development and homeostasis, and its dysregulation contributes to tumor invasion and metastasis, which are key biological hallmarks of tumor aggressiveness^6^. Matrix metalloproteinases (MMPs), a family of zinc-dependent proteases, are secreted by tumor cells and stromal cells^7^ and play crucial roles in remodeling the ECM^8,9^. Although a broad-spectrum inhibitor of MMPs has shown potential efficacy in patients with gastric or GEJ adenocarcinoma, it causes dose-limiting musculoskeletal toxicity, likely due to a lack of specificity^10^.

In addition to matrix remodeling, MMP9 has significant relevance to angiogenesis, the epithelial to mesenchymal transition (EMT) process, and immune suppression^11–14^. Helicobacter pylori infection creates a pro-tumorigenic microenvironment by promoting the activity of MMPs, including MMP9^15,16^, which have been implicated in the pathogenicity and development of gastric adenocarcinoma^17^. MMP9 overexpression is frequently found in gastric adenocarcinoma cells and is associated with the aggressive phenotype and poor prognosis^7,18–22^. In preclinical studies using gastric adenocarcinoma models, MMP9 inhibitors enhanced the cytotoxic effects of chemotherapeutic agents and suppressed distant metastasis^14^, supporting the rationale for combining an MMP9 inhibitor with chemotherapy.

Andecaliximab (ADX, formerly GS-5745) is a recombinant chimeric immunoglobulin G4 monoclonal antibody with high selectivity and affinity for MMP9^23^. In a phase I study, ADX plus modified oxaliplatin, leucovorin, and fluorouracil (mFOLFOX6) was well tolerated and showed promising antitumor efficacy in patients with advanced gastric or GEJ adenocarcinoma^8^. We conducted this phase Ib study including two cohorts, wherein ADX was combined with an oral fluoropyrimidine S-1 plus cisplatin (SP)^24^ or S-1 plus oxaliplatin (SOX)^25,26^ as the first-line treatment for Japanese patients with human epidermal growth factor receptor 2 (HER2)-negative advanced gastric or GEJ adenocarcinoma (ClinicalTrials.gov identifier: NCT02862535).

## Patients and methods

### Study design

This phase Ib, open-label, multicenter study consisted of four cohorts: monotherapy (cohort 1), combination therapy of ADX and S-1 plus platinum (cisplatin in cohort 2, oxaliplatin in cohort 3) in the first-line treatment, and combination therapy of ADX and anti-programmed death-1 (PD-1) antibody nivolumab (cohort 4) (Fig. S1). This report concerns 16 patients enrolled in cohort 2 (combination therapy of ADX and SP) and cohort 3 (combination therapy of ADX and SOX) in patients with HER2-negative gastric or GEJ adenocarcinoma. Results of cohorts 1 and 4 will be reported separately. The study was conducted according to the Declaration of Helsinki and was approved by the local ethics committees/institutional review boards of Cancer Institute Hospital of Japanese Foundation for Cancer Research, National Cancer Center, Aichi Cancer Center, and Osaka University Hospital. All patients provided written informed consent before entering the study. The first patient was screened on September 19, 2016, and the final observation date for the primary end point was October 25, 2019. This study is registered with ClinicalTrials.gov (Identifier: NCT02862535) on August 11th, 2016, and its unique protocol ID is GS-US-296-1884.

### Study treatment

The treatment schedule for each cohort is shown in Fig. S2. The study treatment was continued until disease progression, death, unacceptable toxicity, or withdrawal of consent. Clinical safety and pharmacokinetic data collected from cohort 1 were used to determine the ADX dose for combination therapy.

In cohort 2 (ADX and SP combination therapy), ADX was administered biweekly at a dose of 800 mg via intravenous (IV) infusion for approximately 30 min. Cisplatin was administered at a dose of 60 mg/m^2^ by IV infusion on day eight in five-week intervals. S-1 was administered orally twice daily at 80 mg/day to patients with a body surface area (BSA) < 1.25 m^2^, 100 mg/day to those with a BSA ≥ 1.25 to < 1.5 m^2^, and 120 mg/day to those with a BSA ≥ 1.5 m^2^ for the first 21 days of the 35-day cycle.

In cohort 3 (ADX and SOX combination therapy), ADX was administered at a dose of 1200 mg via IV infusion for approximately 30 min every three weeks. Oxaliplatin was administered by IV infusion at a dose of 100 mg/m^2^ for 2 h on day 1 of each 21-day cycle after completing ADX administration. S-1 at the same dose to cohort 2 was administered orally twice daily. The doses of cisplatin and oxaliplatin were adjusted if the patient’s weight changed by more than 10% of the baseline dosing weight. S-1, cisplatin, and oxaliplatin were obtained from the available commercial supplies at each study site. The dose was reduced on the patient’s condition, the investigator’s discretion, and institutional practice.

### Patient eligibility

The key inclusion criteria included age ≥ 20 years, an Eastern Cooperative Oncology Group performance status (ECOG PS) of ≤ 1, the presence of a histologically confirmed, unresctable advanced or recurrent adenocarcinoma of the stomach or GEJ that had not been treated in the metastatic setting; the presence of a HER2-negative tumor, adequate baseline organ function (within 28 days before day 1 of study treatment). The key exclusion criteria included significant comorbid medical conditions that posed a risk to patient safety or limited study participation, women who were pregnant or breastfeeding, untreated central nervous system metastases, known human immunodeficiency virus and hepatitis B or C infection, history of a concurrent or second malignancy, and. radiotherapy within the previous 28 days. However, patients given palliative radiotherapy to peripheral sites may have entered the study before 28 days had elapsed if patients had recovered from any acute adverse effects.

### Endpoints

The primary objective of this study was to characterize the safety and tolerability of ADX in combination with S-1 plus platinum chemotherapy in Japanese patients with unresectable or recurrent gastric or GEJ adenocarcinoma. The exploratory objective was to assess the therapeutic efficacy of ADX in combination with S-1 plus platinum.

### Safety assessments

Safety was evaluated through clinical laboratory tests, physical examination, 12-lead electrocardiogram analysis, and vital sign measurements. Adverse events (AEs) were assessed according to the Common Terminology Criteria for Adverse Events version 4.03^27^.

### Efficacy assessments

The exploratory efficacy endpoints included the objective response rate (ORR), progression-free survival (PFS), and overall survival (OS). ORR was defined as the proportion of patients with a complete response (CR) or partial response (PR) as the best overall response during ADX therapy, based on Response Evaluation Criteria in Solid Tumors (RECIST) v1.1 guidelines^28^. A two-sided exact 90% confidence interval (CI) for each proportion was calculated using a binomial distribution. PFS was defined as the time interval from the first dose of ADX to the earlier event of the first documentation of definitive disease progression or death from any cause, analyzed using Kaplan‐Meier methods. Definitive disease progression was defined based on RECIST 1.1. OS was defined as the time from the first administration of ADX to death from any cause.

### Ethics committees/institutional review boards

Cancer Institute Hospital of Japanese Foundation for Cancer Research, National Cancer Center, Aichi Cancer Center, Osaka University Hospital.

## Results

### Patient characteristics

Between September 2016 and December 2019, 16 patients with HER2-negative gastric/GEJ adenocarcinoma were enrolled (six patients in the SP combination cohort [cohort 2], and 10 patients in the SOX combination cohort [cohort 3]) (Fig. S3). All 16 patients were suitable for the safety analysis, and their baseline characteristics are summarized in Table 1. The median age was 66 (range = 29–79) years, and most patients were male (69%) and ECOG PS grade 0 (81%). The primary tumor type was gastric in 75% of the patients, and poorly differentiated histology was present in 50% of the patients.

**Table 1:** Baseline patient characteristics.

	Cohort 2	Cohort 3	Total
	(n = 6)	(n = 10)	(n = 16)
Age, median years (range)	68 (57–79)	60 (29–71)	66 (29–79)
Male, n (%)	5 (83.3)	6 (60.0)	11 (68.8)
**ECOG PS, n (%)**			
0	4 (66.7)	9 (90.0)	13 (81.3)
1	2 (33.3)	1 (10.0)	3 (18.8)
**Primary tumor site, n (%)**			
Gastric	5 (83.3)	7 (70.0)	12 (75.0)
Proximal	1 (16.7)	3 (30.0)	4 (25.0)
Distal	0	0	0
Other	4 (66.7)	4 (40.0)	8 (50.0)
GEJ	1 (16.7)	3 (30.0)	4 (25.0)
**Differentiation, n (%)**			
Well differentiated	0	2 (20.0)	2 (12.5)
Moderately differentiated	2 (33.3)	4 (40.0)	6 (37.5)
Poorly differentiated	4 (66.7)	4 (40.0)	8 (50.0)

The safety analysis set includes subjects who received at least one dose of ADX.

*ECOG PS* Eastern Cooperative Oncology Group performance status, *GEJ* gastroesophageal junction.

### Study drug exposure

A total of 16 patients received at least one dose of the study drug in cohorts of combination therapy with ADX and S-1 plus platinum. The median duration of exposure to ADX was 35 (interquartile range = 23–51) weeks, with 18 (interquartile range = 6–32) weeks in cohort 2 and 57 (interquartile range = 47–69) weeks in cohort 3. The median total number of ADX doses received per patient was 12 (interquartile range = 9–17), with 10 (interquartile range = 4–17) in cohort 2 and 18 (interquartile range = 16–22) in cohort 3. The median duration of exposure to cisplatin and oxaliplatin was 20 and 32 weeks, respectively, and the median total number of cisplatin and oxaliplatin doses received per patient was 5 and 10, respectively, in cohorts 2 and 3.

### Safety

Treatment-emergent adverse events (TEAEs) of any grade were reported in all 16 patients in cohort 2 and cohort 3 (Table 2). In total, the most common TEAEs were peripheral sensory neuropathy (69%), anorexia (63%), nausea (56%), and decreased neutrophil counts (44%). Decreased appetite and nausea (each five patients, 83.3%) and diarrhea (four patients, 66.7%) were reported in cohort 2. Peripheral sensory neuropathy (10 patients, 100%), decreased appetite and platelet counts (each 5 patients, 50.0%), and nausea, constipation, and decreased white blood cell counts (each 4 patients, 40%) were observed in cohort 3. Two patients (33.3%) in cohort 2 and nine patients (90.0%) in cohort 3 had TEAEs that the investigator considered to be related to ADX. The main AEs attributed to ADX were stomatitis (two patients, 33.3%) in cohort 2 and constipation, increased amylase, and decreased neutrophil and white blood cell counts (each two patients, 20%) in cohort 3. Six patients (100%) in cohort 2 and nine subjects (90%) in cohort 3 had AEs attributed to S-1. Five subjects (83.3%) in cohort 2 and all 10 patients (100%) in cohort 3 had AEs attributed to cisplatin and oxaliplatin.

**Table 2:** Treatment-emergent adverse events (TEAEs) of any grade observed in ≥ 15% of all patients.

	Cohort 2	Cohort 3	Total
	(n = 6)	(n = 10)	(n = 16)
TEAEs of any grade	6 (100.0)	10 (100.0)	16 (100.0)
Peripheral sensory neuropathy	1 (16.7)	10 (100.0)	11 (68.8)
Decreased appetite	5 (83.3)	5 (50.0)	10 (62.5)
Nausea	5 (83.3)	4 (40.0)	9 (56.3)
White blood cell count decreased	3 (50.0)	4 (40.0)	7 (43.8)
Constipation	2 (33.3)	4 (40.0)	6 (37.5)
Diarrhea	4 (66.7)	2 (20.0)	6 (37.5)
Neutrophil count decreased	3 (50.0)	3 (30.0)	6 (37.5)
Platelet count decreased	1 (16.7)	5 (50.0)	6 (37.5)
Stomatitis	3 (50.0)	3 (30.0)	6 (37.5)
Fatigue	2 (33.3)	2 (20.0)	4 (25.0)
Aspartate aminotransferase increased	0	3 (30.0)	3 (18.8)
Insomnia	3 (50.0)	0	3 (18.8)
Pyrexia	2 (33.3)	1 (10.0)	3 (18.8)
Rash maculo-popular	2 (33.3)	1 (10.0)	3 (18.8)
Upper respiratory tract infection	1 (16.7)	2 (20.0)	3 (18.8)
Vascular pain	0	3 (30.0)	3 (18.8)
Vomiting	2 (33.3)	1 (10.0)	3 (18.8)

The safety analysis set includes subjects who received at least one dose of ADX.

Treatment-emergent adverse events (TEAEs) are AEs with onset dates on or after the first dose of study drug ADX and up to 30 days after permanent withdrawal of ADX.

Grade 3 or higher TEAEs were reported in five patients (83.3%) in cohort 2 and five patients (50.0%) in cohort 3 (Table 3). The grade 3 or higher AEs were mainly decreased neutrophil counts in six patients (38%), three patients in cohort 2 and three patients in cohort 3. Other AEs included increased amylase, increased CPK, abnormal hepatic function, anorexia, hyperglycaemia, hyponatraemia, anemia, stomatitis, and peripheral sensory neuropathy (each one patient, 6.2%) in all 16 patients. The grade 3 or higher AEs attributed to ADX were stomatitis and hepatic function abnormal (each one patient, 16.7%) in cohort 2, and decreased neutrophil counts (two patients, 20.0%), increased amylase (one patient, 10.0%), and increased blood creatine phosphokinase (one patient, 10.0%) in cohort 3.

**Table 3:** Treatment-emergent adverse events (TEAEs) with grade ≥ 3.

	Cohort 2	Cohort 3	Total
	(n = 6)	(n = 10)	(n = 16)
With grade 3 or higher	5 (83.3)	5 (50.0)	10 (62.5)
Neutrophil count decreased	3 (50.0)	3 (30.0)	6 (37.5)
Amylase increased	0	1 (10.0)	1 (6.3)
Blood creatine phosphokinase increased	0	1 (10.0)	1 (6.3)
Bile duct stenosis	0	1 (10.0)	1 (6.3)
Hepatic function abnormal	1 (16.7)	0	1 (6.3)
Decreased appetite	1 (16.7)	0	1 (6.3)
Hyperglycemia	1 (16.7)	0	1 (6.3)
Hyponatremia	1 (16.7)	0	1 (6.3)
Anemia	1 (16.7)	0	1 (6.3)
Stomatitis	1 (16.7)	0	1 (6.3)
Peripheral sensory neuropathy	0	1 (10.0)	1 (6.3)
Pneumothorax	1 (16.7)	0	1 (6.3)

The safety analysis set includes subjects who received at least one dose of ADX.

TEAEs are AEs with onset dates on or after the first dose of study drug ADX and up to 30 days after permanent withdrawal of ADX.

In cohort 3, eight patients (80.0%) in cohort 3 had AEs leading to dose modifications or the temporary interruption of ADX treatment, and the only AE reported in more than one patient was a decreased neutrophil count (three patients, 30.0%). Five patients (83.3%) in cohort 2 and eight patients (80.0%) in cohort 3 had an AE that resulted in dose modifications or the temporary interruption of S-1 treatment, and the only AE related to S-1 reported in more than one patient was a decreased neutrophil count (3 subjects, 30.0%) in cohort 3. The AEs attributed to platinum reported in more than one patient were grade 1 malaise and grade 2 upper respiratory tract infection (each one patient, 16.7%) in the cisplatin group (cohort 2) and peripheral sensory neuropathy (six patients, 60.0%), decreased neutrophil counts (three patients, 30.0%), and decreased platelet counts (two patients, 20.0%) in in the oxaliplatin group (cohort 3). No patients experienced AEs leading to discontinuation of the study drug, and no AEs led to death.

Serious adverse events (SAEs) were reported in two patients (33.3%) in cohort 2 and one patient (10.0%) in cohort 3. In cohort 2, two patients (33.3%) had grade 3 abnormal hepatic function that was judged by the investigator to be related to ADX and S-1 in each one patient, respectively. In cohort 3, one patient had grade 3 bile duct stenosis. There were no SAEs related to cisplatin or oxaliplatin.

### Efficacy

**Figure 1:**
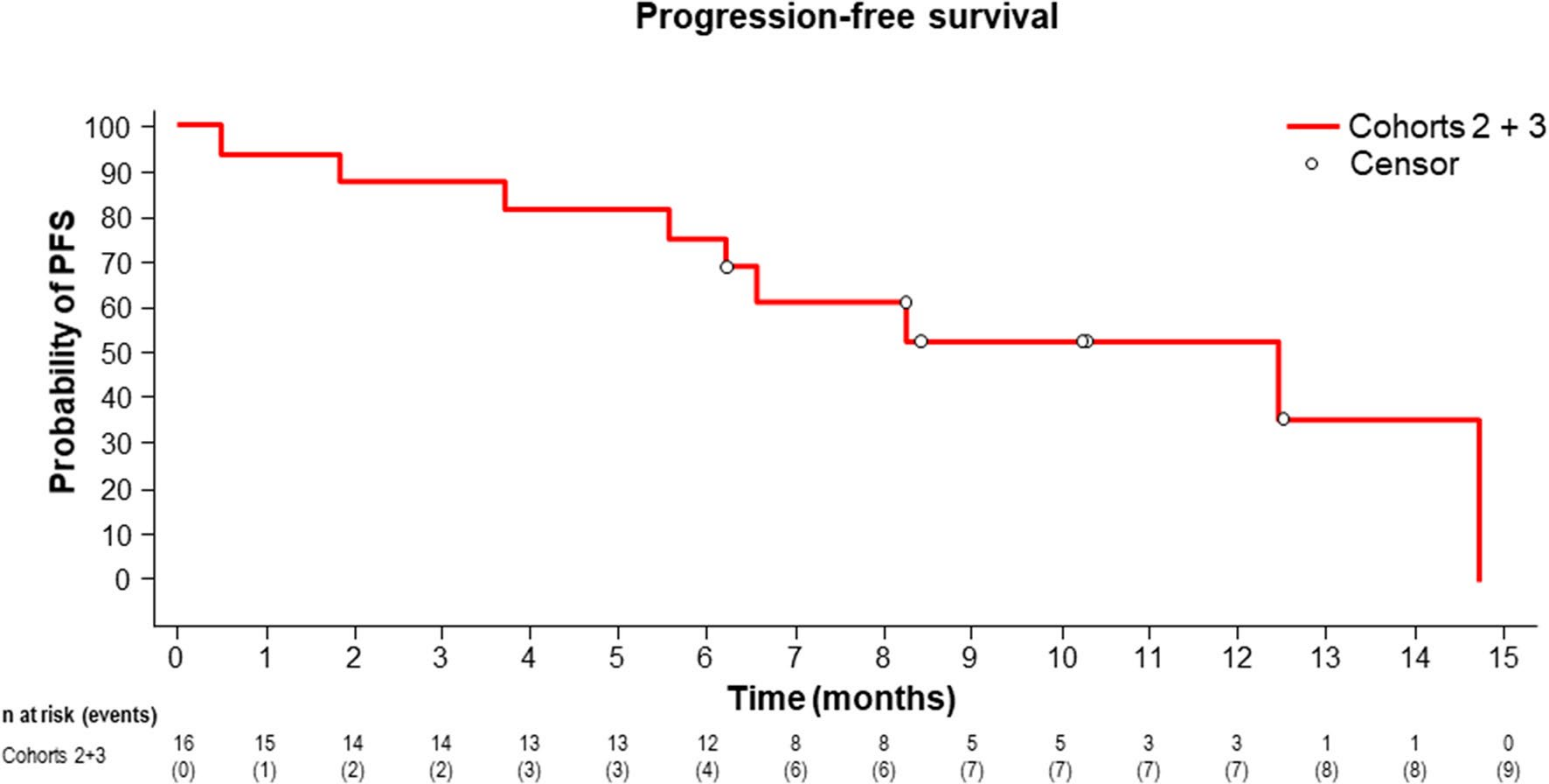
Kaplan–Meier curve of PFS when cohorts 2 and 3 were combined. Kaplan–Meier curve was estimated from cut-off data of December 2018. The median PFS was 12.5 months (90% CI 5.6–14.7). After follow up at the data cut-off date (December, 2019), the median PFS was 11.9 months (90% CI 5.6–16.6).

**Figure 2:**
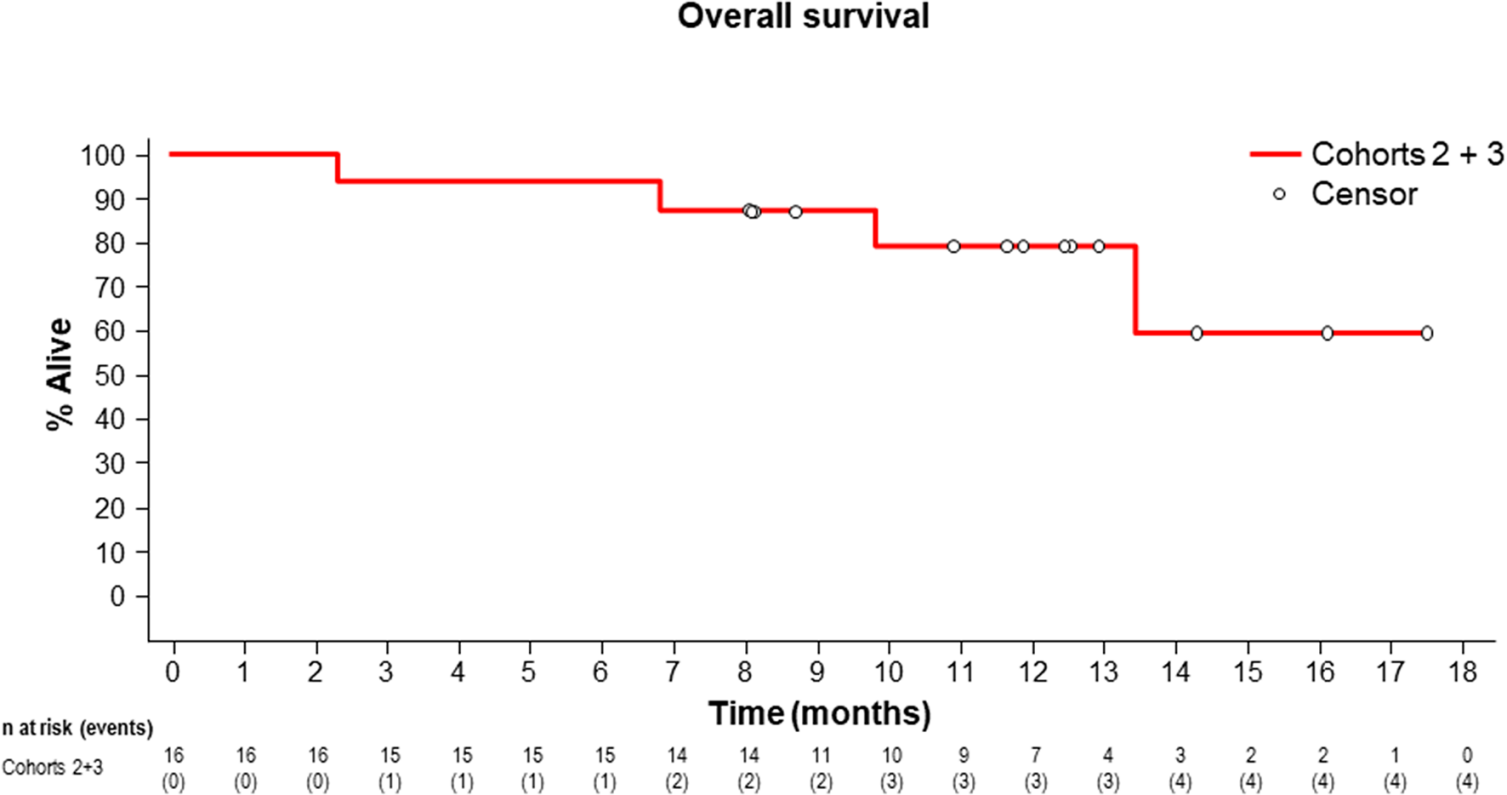
Kaplan–Meier curve of OS when cohorts 2 and 3 were combined.

In 11 patients with measurable target lesions, including five patients in cohort 2 and six patients in cohort 3, the ORR was 73% (8/11), based on the investigator’s evaluation (Table 4). There were one CR and two PR in cohort 2 and five PR in cohort 3. When cohorts 2 and 3 were combined, the median PFS was 11.9 months (90% CI 5.6–16.6) (Fig. 1). The Kaplan–Meier estimate of the median PFS was 4.6 (90% CI 0.5–14.7) months in cohort 2 (Fig. S4) and 16.6 (90% CI 6.2–not estimated) months in cohort 3 (Fig S5). The estimated PFS rates at 3 and 6 months were 66.7% and 33.3%, respectively, in cohort 2 and 100.0% and 100.0%, respectively, in cohort 3. The median OS for the combined cohorts was not reached (Fig. 2). The Kaplan–Meier estimate of the median OS was not reached (90% CI 2.3–not estimated) in cohort 2 (Fig. S6) and not reached (90% CI 9.8–not estimated) in cohort 3 (Fig. S7). The estimated OS rates at 12 and 18 months were 66.7% and 50.0%, respectively, in cohort 2, and 90.0% and 67.5%, respectively, in cohort 3.

**Table 4:** ORR in patients with measurable lesions.

	Cohort 2	Cohort 3	Total
	(n = 5)	(n = 6)	(n = 11)
**Best overall response**			
Complete response (CR)	1 (20.0)	0	1 (9.1)
Partial response (PR)	2 (40.0)	5 (83.3)	7 (63.6)
Stable disease (SD)	0	1 (16.7)	1 (9.1)
Progressive disease (PD)	2 (40.0)	0	2 (18.2)
Not-evaluable (NE)	0	0	0
Objective response rate, % (90% CI)	60.0 (18.9, 92.4)	83.3 (41.8, 99.1)	72.7 (43.6, 92.1)
Responders (CR + PR), n (%)	3 (60.0)	5 (83.3)	8 (72.7)
Non-responder (SD + PD + NE), n (%)	2 (40.0)	1 (16.7)	3 (27.3)

The safety analysis set includes subjects who received at least one dose of ADX.

Objective response rate is defined as (number of responders)/(number of responders + number of non-responders).

Two-sided exact 90% confidence interval (CI) for proportions was determined using the binomial distribution.

## Discussion

Based on the potential clinical activity of ADX in patients with advanced gastric or GEJ adenocarcinoma^8^, we conducted a phase 1b study to evaluate the safety and tolerability of ADX in four cohorts: monotherapy (cohort 1), combination therapy with ADX and S-1 plus platinum as a first-line treatment (cohort 2 and 3), and combination therapy with ADX and nivolumab (cohort 4). Here, we present data from 16 patients with HER2-negative gastric or GEJ adenocarcinoma enrolled in cohort 2 and 3. The results from cohorts 1 and 4 have been reported elsewhere^29,30^.

The combination of doublet platinum plus fluoropyrimidine is the standard backbone regimen for patients with advanced gastric or GEJ adenocarcinoma in Western^3^ and Asian countries^4,31^. In Asian countries, S-1 has been adopted as an alternative to infusional fluoropyrimidine due to the convenience of oral administration^4,31,32^. Since platinum cisplatin and oxaliplatin have similar efficacy^33^, S-1 plus cisplatin (SP) or oxaliplatin (SOX) is the preferred first-line chemotherapy regimens^4,24–26,31^. In this study, there were no AEs leading to discontinuation of the study drug, and all patients discontinued study treatment due to disease progression without grade 5 AEs. The most common TEAEs were peripheral sensory neuropathy, anorexia, nausea, and leukopenia. The safety profile of ADX combined with SP or SOX was similar to the previously reported toxicity profile for SP or SOX chemotherapy alone in first-line treatment^25,26^, consistent with a previous study showing that there were no meaningful differences in AEs between mFOLFOX6 treatments with and without ADX^34^. Thus, the combination of ADX with SP or SOX appeared to be well tolerated without new or unexpected toxicity profiles.

The preliminary efficacy data regarding the addition of ADX to S-1 plus platinum revealed a median PFS of 11.9 months (90% CI 5.6–16.6), with an ORR of 73% in patients with measurable target lesions. In two phase III studies comparing SOX with SP, the median PFS and ORR were 5.5–5.6 months and 56–58% for SOX and 5.4–5.7 months and 52–60% for SP, respectively^25,26^, suggesting the potential for additional improvement with ADX compared to S-1 plus platinum combination chemotherapy as a first-line treatment in patients with advanced gastric or GEJ adenocarcinoma. The addition of ADX showed a more favorable efficacy trend when combined with SOX (cohort 3) than SP (cohort 2); however, this is not a comparable result due to the small number of patients, independent cohorts, and biased population (e.g., the higher median age and poorly differentiated histology in cohort 2). Based on the results showing no survival benefit of OS and PFS in a phase III study comparing between mFOLFOX6 with and without ADX as a first-line treatment for unselected patients with HER2-negative advanced gastric or GEJ adenocarcinoma^34^, this study was closed. However, the ORR in the mFOLFOX6 with ADX group was higher than that in the mFOLFOX6 without ADX group (51% vs. 41%, P = 0.049), indicating that a subset of patients is likely to benefit from ADX plus chemotherapy. Two recent phase II studies showed impressive results of agents targeting claudin-18 isoform 2 (zolbetuximab) and fibroblast growth factor receptor-2 isoform IIIb (bemarituzumab)^35^ in combination with first-line chemotherapy for patients with HER2-negative gastric cancer. These studies also demonstrated proof-of-concept, suggesting that biomarker selection is required to improve the efficacy of molecular targeted agents^36^. Therefore, identification of predictive biomarkers is vital to improve the clinical outcome of ADX plus chemotherapy.

MMP9 regulates growth factors, cytokines, and chemokines and promotes an immune suppressive tumor microenvironment via matrix remodeling, tumor infiltration of T cells, and recruitment and activation of myeloid-derived suppressor cells^8,9,11–13,37–40^. Consistent with preclinical findings suggesting that dual blockade of MMP9 and programmed death ligand 1 (PD-L1) may lead to an improved antitumor immune response^23^, the outcomes in cohort 4 treated with ADX plus nivolumab have been reported elsewhere, with promising clinical activity^30^. Although there were no statistically significant differences, treatment with ADX plus nivolumab resulted in favorable disease control rate (30.6% vs. 23.6%) and prognosis (median OS, 7.1 months vs. 5.9 months), compared with nivolumab alone, in a randomized phase II study^41^. Recently, the combination of nivolumab and chemotherapy represents a new standard in first-line treatment for patients with HER2-negative gastric cancer^42^. Considering a possible synergistic effect of ADX and nivolumab, the addition of ADX in combination with nivolumab plus chemotherapy may be a potent regimen.

In gastric adenocarcinoma, MMP9 may be implicated in tumor progression and has potential use as a diagnostic and prognostic biomarker^7,18–22,43^. MMP-9 interacts with cell surface proteins, including the cholangiocarcinoma cluster of differentiation 44 (CD44)^44^ which is a cell surface marker of gastric cancer stem-like cells (CSCs)^45^ and imparts gastric cancer CSC properties by promoting the synthesis of intracellular reduced glutathione^46^. Furthermore, preclinical studies of gastric adenocarcinoma have shown that MMP-9 drives angiogenesis^37^ and distant metastasis through EMT properties induced by the phosphoinositide 3-kinase (PI3K)-AKT-snail signaling axis^14^. Thus, MMP9 remains an attractive therapeutic target for gastric or GEJ adenocarcinoma. Further understanding of MMP9 biology and ways to modulate MMP9 activity might enable the development of effective therapy in gastric cancer in the future^47,48^.

## Conclusion

ADX in combination with S-1 plus platinum demonstrated promising clinical activity and well-tolerated AEs, as well as toxicity profiles consistent with those previously reported with chemotherapy alone, in patients with advanced gastric or GEJ adenocarcinoma. However, survival benefits were not shown in a randomized phase III study of ADX combined with a first-line mFOLFOX6 treatment. Thus, the development of ADX in oncology was discontinued, and this study was closed.

## Supplementary Information

Supplementary Legends.

Supplementary Figure S1.

Supplementary Figure S2.

Supplementary Figure S3.

Supplementary Figure S4.

Supplementary Figure S5.

Supplementary Figure S6.

Supplementary Figure S7.
