# Supplementary material for: A phase 1b study of andecaliximab in combination with S-1 plus platinum in Japanese patients with gastric adenocarcinoma
Source: Sci Rep. 2022 Jun 30;12:11007. doi: 10.1038/s41598-022-13801-1 (PMC9246925; doi:10.1038/s41598-022-13801-1)
Supplement: Supplementary file 1 — Supplementary Legends. [file 41598_2022_13801_MOESM1_ESM.docx]

**A List of Supporting Information**

**Supplementary Figure S1:** GS-US-296-1884 study schema.

**Supplementary Figure S2:** Treatment schedule for each cohort.

**Supplementary Figure S3:** Patient disposition in the study.

**Supplementary Figure S4:** The Kaplan–Meier curves of PFS in the cohort 2.

Kaplan-Meier curve was estimated from cut-off data of December 2018. After follow up at the data cut-off date (December, 2019), the median PFS was 4.6 (90% CI = 0.5–14.7) months.

**Supplementary Figure S5:** The Kaplan–Meier curves of PFS in the cohort 3.

Kaplan-Meier curve was estimated from cut-off data of December 2018. After follow up at the data cut-off date (December, 2019), the median PFS was 16.6 (90% CI = 6.2–not estimated) months.

**Supplementary Figure S6:** The Kaplan–Meier curves of OS in the cohort 2.

Kaplan-Meier curve was estimated from cut-off data of December 2018. After follow up at the data cut-off date (December, 2019), the median OS was not reached (90% CI = 2.3–not estimated).

**Supplementary Figure S7:** The Kaplan–Meier curves of OS in the cohort 3.

Kaplan-Meier curve was estimated from cut-off data of December 2018. After follow up at the data cut-off date (December, 2019), the median OS was not reached (90% CI = 9.8–not estimated).
