# Supplementary figures and images for: A phase 1b study of andecaliximab in combination with S-1 plus platinum in Japanese patients with gastric adenocarcinoma
Source: Sci Rep. 2022 Jun 30;12:11007. doi: 10.1038/s41598-022-13801-1 (PMC9246925; doi:10.1038/s41598-022-13801-1)

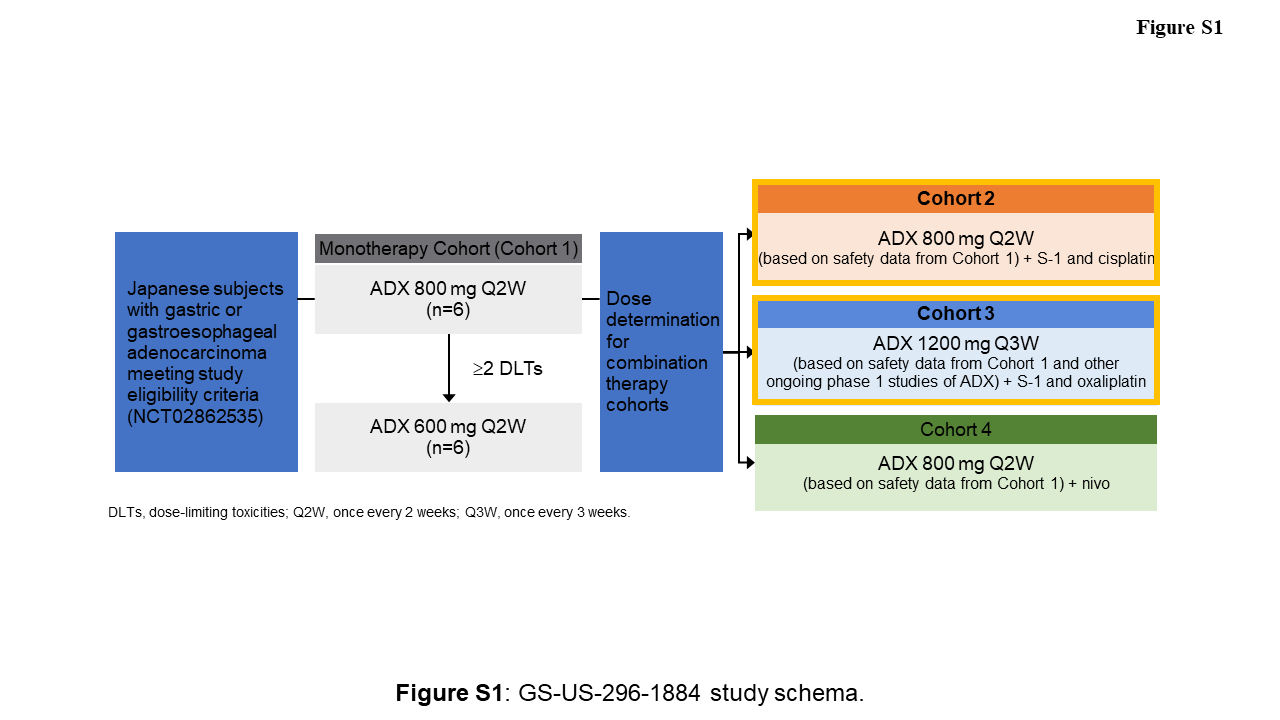

Supplement: Supplementary file 2 — Supplementary Figure S1. [file 41598_2022_13801_MOESM2_ESM.tif]

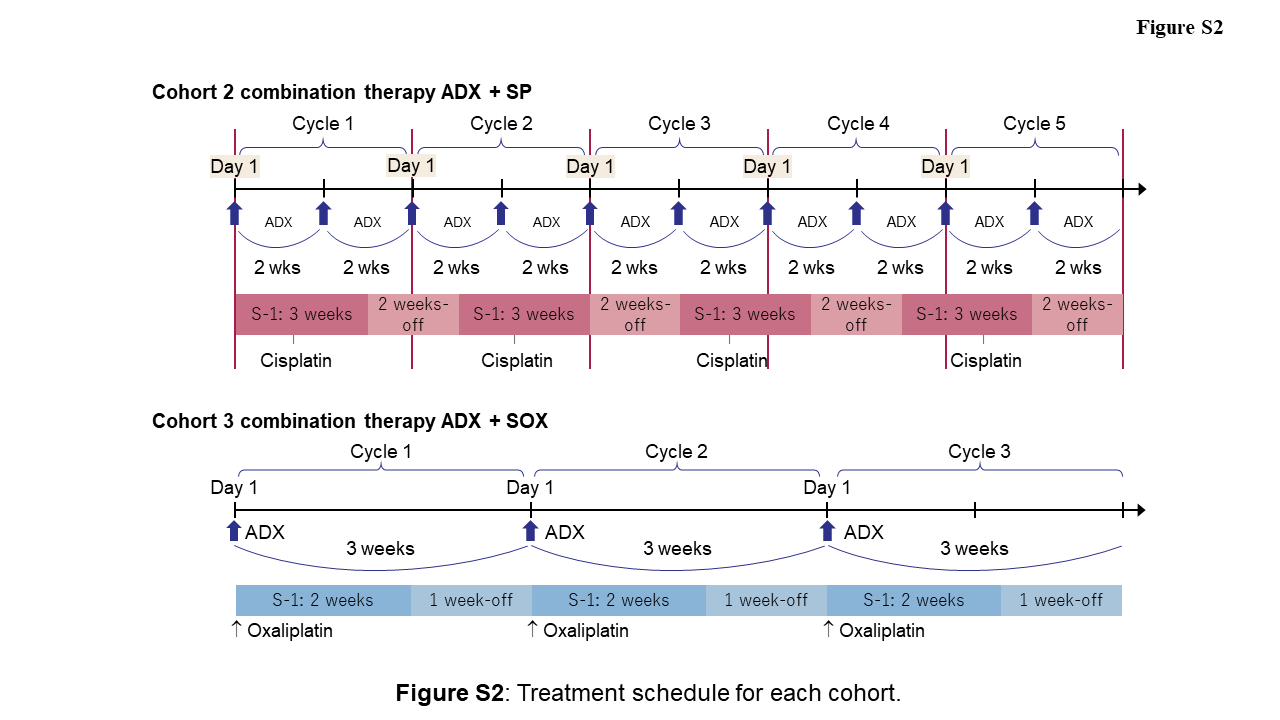

Supplement: Supplementary file 3 — Supplementary Figure S2. [file 41598_2022_13801_MOESM3_ESM.tif]

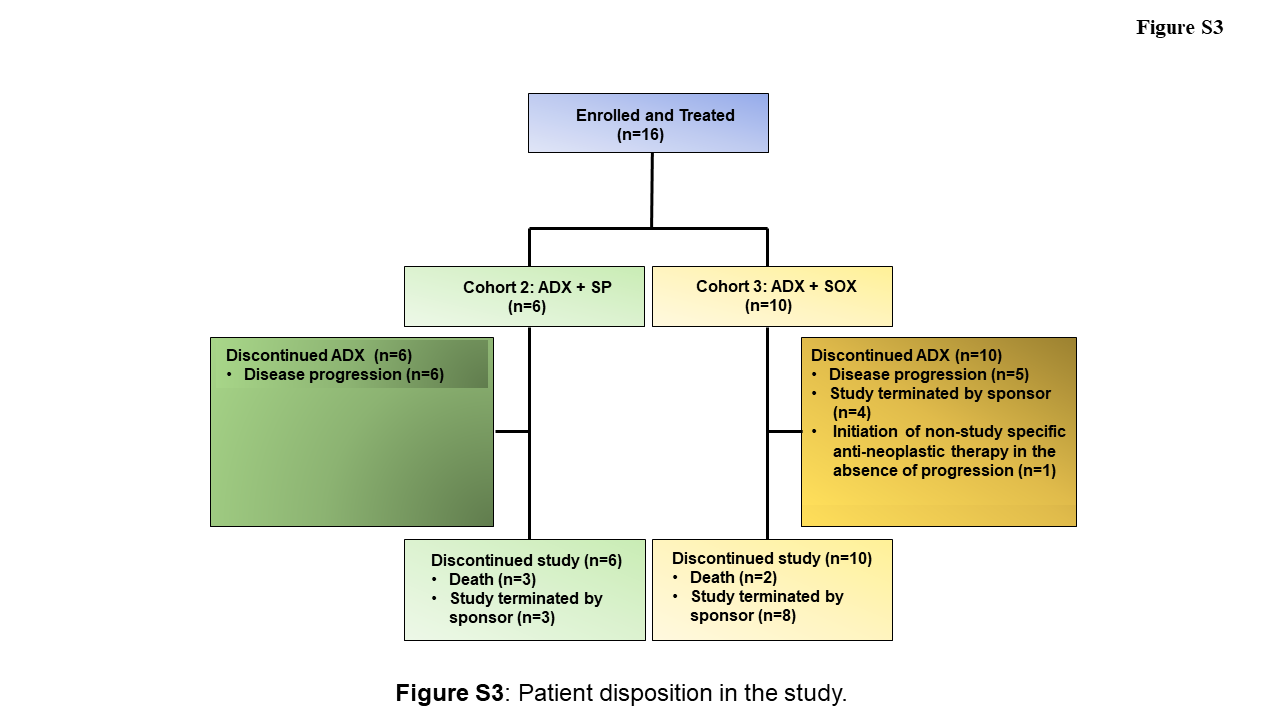

Supplement: Supplementary file 4 — Supplementary Figure S3. [file 41598_2022_13801_MOESM4_ESM.tif]

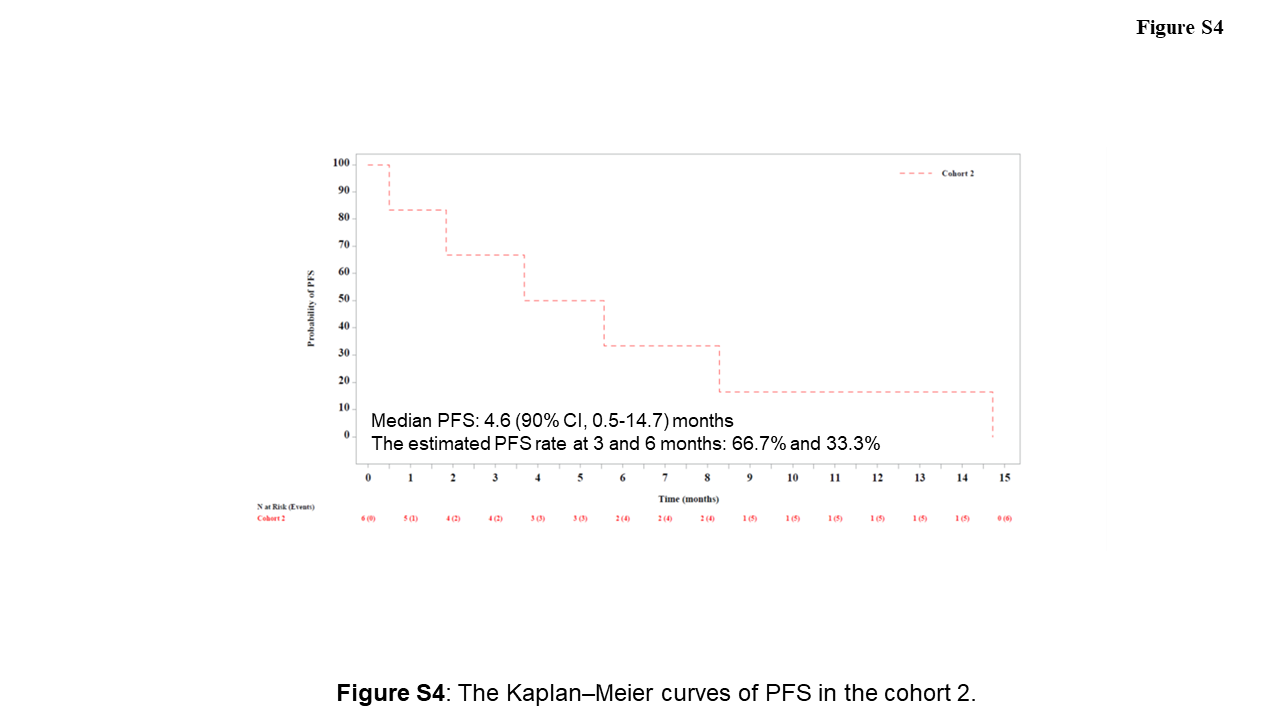

Supplement: Supplementary file 5 — Supplementary Figure S4. [file 41598_2022_13801_MOESM5_ESM.tif]

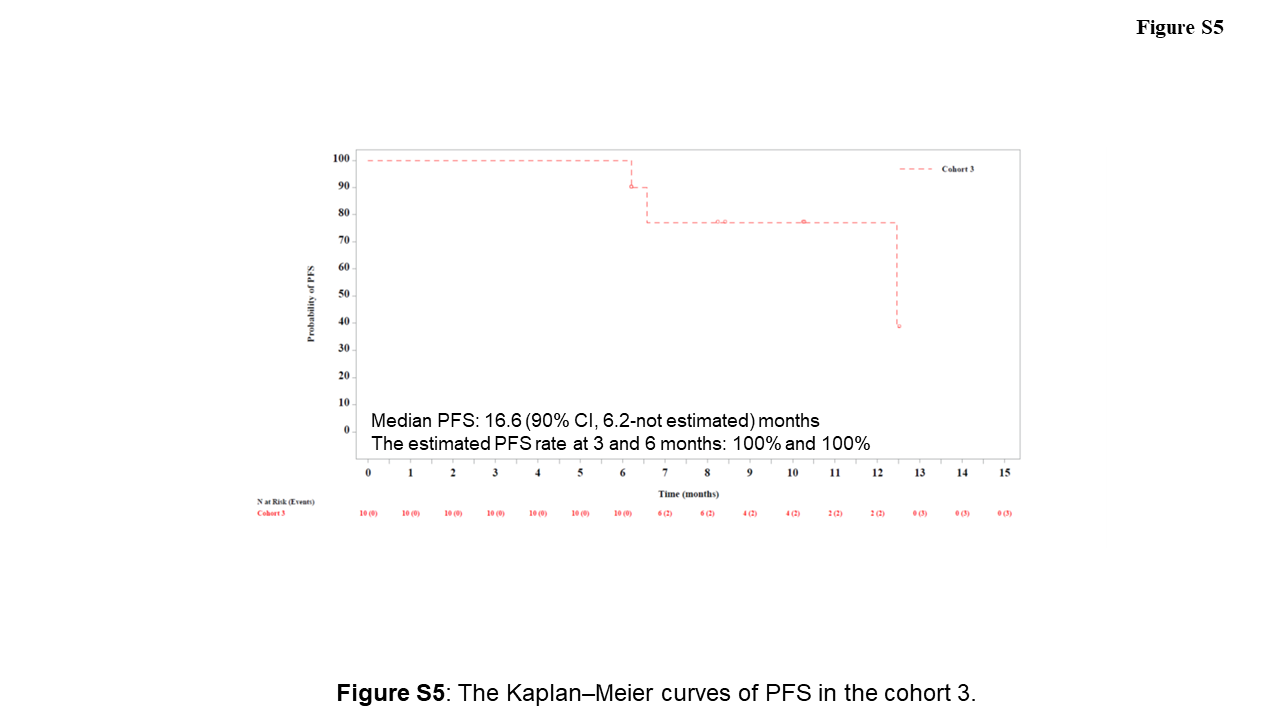

Supplement: Supplementary file 6 — Supplementary Figure S5. [file 41598_2022_13801_MOESM6_ESM.tif]

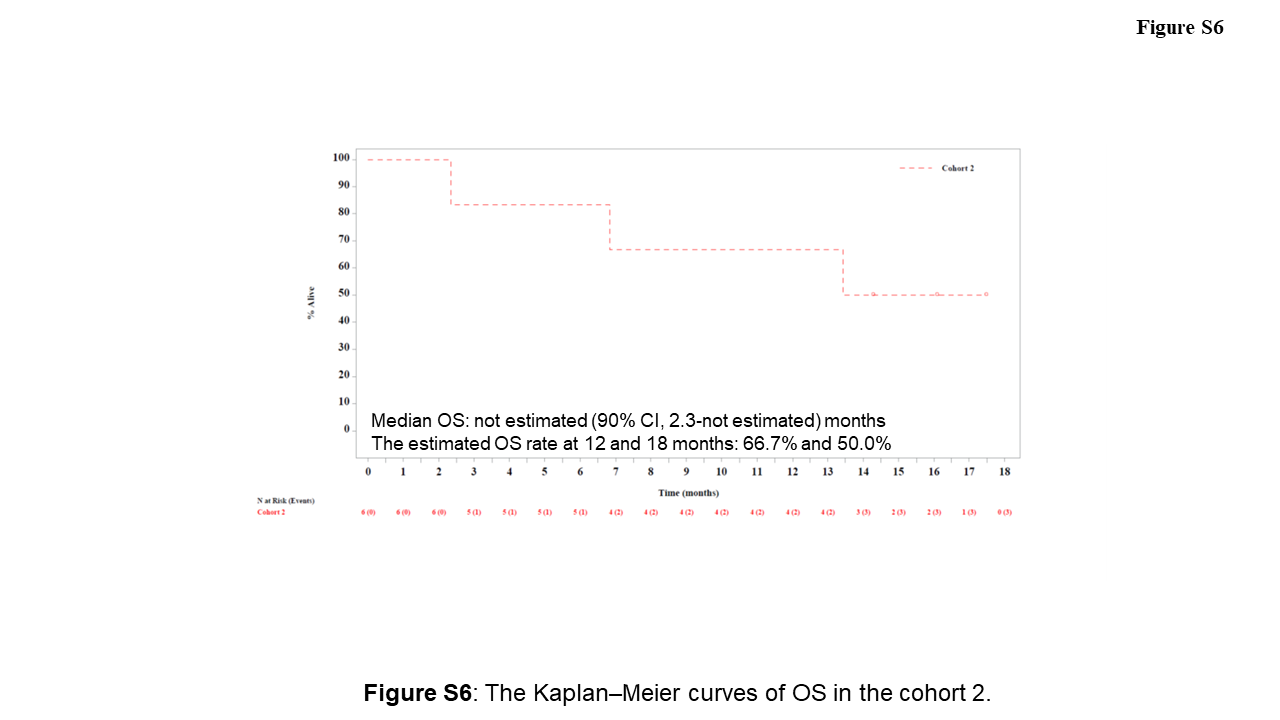

Supplement: Supplementary file 7 — Supplementary Figure S6. [file 41598_2022_13801_MOESM7_ESM.tif]

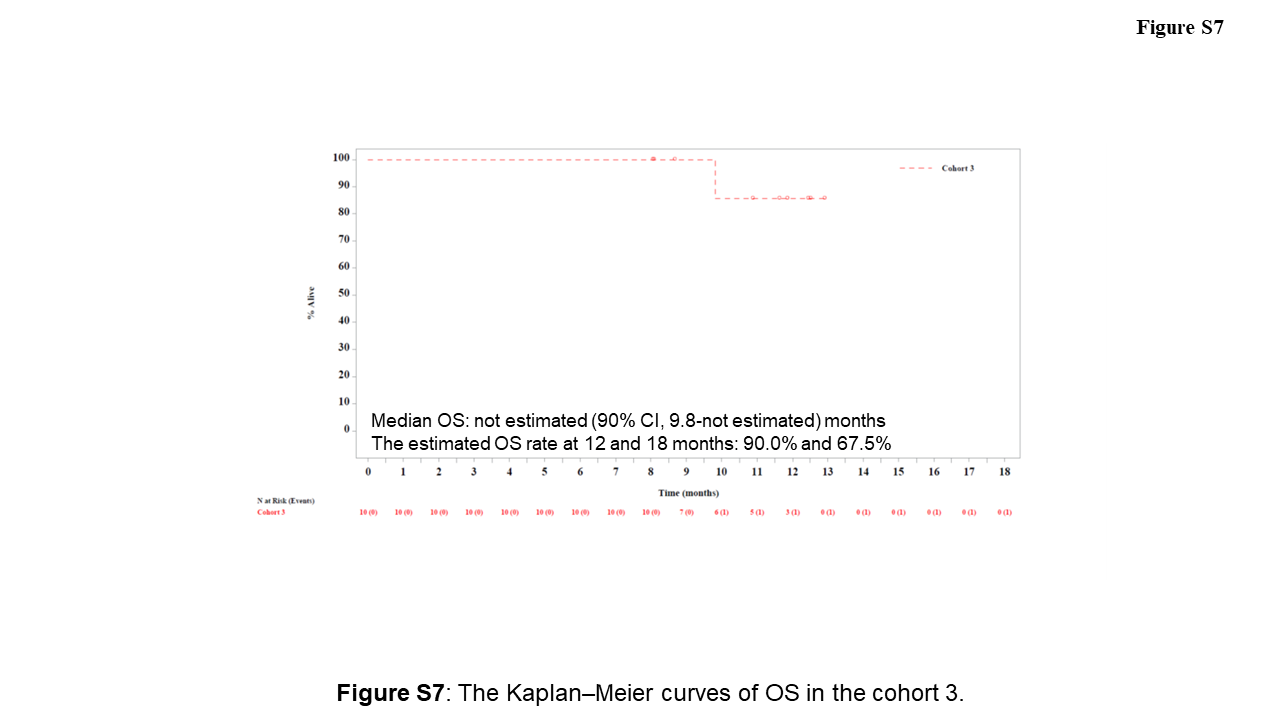

Supplement: Supplementary file 8 — Supplementary Figure S7. [file 41598_2022_13801_MOESM8_ESM.tif]
